# Pterygoid implant: extensometric and photoelastic analysis of a maxillary rehabilitation model

**DOI:** 10.1590/1807-3107bor-2025.vol39.030

**Published:** 2025-03-10

**Authors:** Daniel Henrique KOGA, Marcos Martins CURI, Joel Ferreira SANTIAGO, Aldieris Alves PESQUEIRA, Wagner José Sousa CARVALHO, Márcio CAMPANER, Camila Lopes CARDOSO

**Affiliations:** (a) Hospital Santa Catarina, Department of Oral Surgery, São Paulo, SP, Brazil.; (b) Universidade de São Paulo – USP, Ribeirão Preto Dental School, Department of Prosthodontics, Ribeirão Preto, SP, Brazil.; (c) Univeridadde Estadual Paulista – Unesp, Araçatuba School of Dentistry, Department of Dental Materials and Prosthodontics, Araçatuba, SP, Brazil.; (d) Universidade de São Paulo – USP, School of Public Health, São Paulo, SP, Brazil.; (e) Universidade de São Paulo – USP, Bauru Dental School Department of Surgery, Stomatology, Pathology and Radiology, Bauru, SP, Brazil.

**Keywords:** Implants, Dental Implants, Maxilla, Pterygoid Implant, Sphenoid Bone

## Abstract

Pterygoid implants have been demonstrated to have a high success rate. Nevertheless, there are few biomechanical tests to evaluate the tensile forces induced by force dissipation in peri-implant tissues. This study employed photoelasticity and extensometry to demonstrate and compare the biomechanical behavior of non-axial implants in a pterygoid model and a conventional model of oral rehabilitation, thus allowing for qualitative and quantitative analysis. Two models received an implant measuring 3.75 x 13 mm in the canine pillar at a 90 ° angle to the Frankfurt plane. In the control group, an implant with a diameter of 3.75 mm and a length of 11 mm was placed in the maxillary tuberosity parallel the medial implant. In the study group, an implant with a diameter of 3.75 mm and a length of 11 mm was installed with an angulation of 45 degrees in the antero-posterior direction and 15 degrees in the buccal-palatal direction, with apical anchorage in the pterygoid process of the sphenoid bone. In the extensometric analysis, the models were subjected to five cycles of repeated axial tensile loading (100 N) at a rate of 0.5 mm/min. A computer was connected to the amplifier in order to record the output signal of the polyurethane surface, and the acquisition system software was employed to record the data. The data were analyzed in accordance with data distribution, as determined by the Shapiro-Wilk test and equality of variance. Subsequently, the data were classified according to the variables. The Student’s t-test was employed when normal distribution of variances was identified, whereas the Mann-Whitney U test was utilized for data with non-normal distribution. A 5% significance level was employed. In the photoelastic analysis, replicas of both configurations were produced using photoelastic resin. The models were subjected to a single axial loading cycle, with a load of 100 N applied at a rate of 0.5 mm/min, and the resulting stress was observed under a circular polariscope. Photographs were taken at two time points: before and after loading. These images were then processed by the same operator using a computer graphics program, allowing for a more straightforward analysis of stress distribution. This was achieved by the formation of isochromatic fringes. The results of the strain gauge analysis revealed no statistically significant differences between the two groups (p = 0.37) or between the anterior (p = 0.08) and posterior (p = 0.74) implants. The photoelasticity analysis revealed the presence of high-intensity isochromatic fringes at the apex of the axial implant in the control model, as well as in the cervical-distal and apical regions of the pterygoid implant, where a high concentration was also observed. Although no statistically significant results were obtained from the quantitative analysis, our findings suggest that the favorable outcomes observed in the clinical studies are due to the high force dissipation observed in the pterygoid plate, which is composed of dense cortical bone.

## Introduction

Since the introduction of the osseointegration concept by Brånemark,^1^ osseointegrated implants have been successfully employed in the treatment of partial or complete edentulous jaws. Nevertheless, the oral rehabilitation of a patient with atrophic maxilla represents a significant challenge in implant-supported rehabilitations.^2-7^ The placement of implants in the posterior region is often unfeasible due to the atrophy of the alveolar process, the poor bone quality, and the presence of maxillary sinus pneumatization.^5,7-11^

In order to address these limitations, reconstructive procedures utilizing grafts or biomaterials have been proposed as a potential solution.^6,12-14^ The surgical techniques of sinus lifting and inlay and onlay grafts have been well documented in the literature as a means of addressing maxillary atrophy. Nevertheless, this requires the implementation of supplementary surgical techniques, which are associated with a high risk of complications, extended treatment periods and, consequently, increased financial expenditure.^5-6,8,11,15-18^

The placement of implants in the maxillary pillars is a simple technique in the treatment of maxillary atrophy and reduces morbidity.^8,13,16,19-22^ The pterygoid technique, first described by Tulasne in 1989^23^, is based on the implantation of an osseointegrated device within the pillar consisting of the maxillary tuberosity, the palatine bone, and the pterygoid process of the sphenoid bone. It allows anchorage into a greater cortical bone surface, thereby providing high stability and good support for an implant supported prosthesis without a distal cantilever. Furthermore, the technique has been highly successful with a 10-year cumulative survival rate of 92.5%.^3,5-6,24-27^

Laboratory and clinical research indicates that the success and longevity of dental implants can be influenced by biomechanical factors in the majority of cases.^15,28-32^ Techniques such as photoelastic stress analysis, finite element stress analysis, mathematical calculations, and strain gauge analysis have been widely described and effectively used to assess the biomechanical behavior of loads on implants, bone, and prosthetic compounds.^15,28-34^ The technique of photoelasticity allows a visual (qualitative) evaluation of the photographs taken under polariscope whereas extensometry enables a numerical (quantitative) analysis.

Nevertheless, there is a paucity of literature on the biomechanical analysis of a pterygoid implant in a maxillary rehabilitation model. Based on a previous clinical study, our paper describes the mechanical behavior of dental implants anchored in the pterygoid plate using photoelastic analysis and extensometry.^6^The hypothesis of this study is that the tests applied will demonstrate that the mechanical behavior of this type of rehabilitation is acceptable.

## Methods

### Experimental design

Two polyurethane skulls (Nacional Ossos, Jaú, Brazil) were obtained and modified to create replicas. These replicas were used to established guidelines for the installation of dental implants. (Sistema Nacional de Implantes, São Paulo, Brazil). Both models received external hexagonal implants, with a hybrid macrogeometry (cylindrical body and conical apex) measuring 3.75 x 13 mm in the canine pillar at an angle of 90 degrees to the Frankfurt plane. The distal implants were inserted in two configurations, which are outlined below:

Control group: A 3.75 x 11 mm implant was placed in the maxillary tuberosity parallel position to the medial implant.

Pterygoid (study) group: A 3.75 x 15 mm implant was installed in the maxillary tuberosity with an angulation of 45 degrees in the antero-posterior direction and 15 degrees in the buccal-palatal direction according to the Frankfurt plane. The implant was placed with apical anchorage in the pterygoid process of the sphenoid bone.

The models were placed on a flat surface and a bubble level tool was used to verify the parallelism with the Frankfurt plane. A straight line was drawn at a 90-degree angle from the bubble level tool and used as a reference point for the installation of the anterior and maxillary implants. In the pterygoid region, a line was traced at 45 degrees with the Frankfurt plane. The same tools were employed in the lateral plane, whereby the crest was used as a reference to determine 15 degrees, thus reaching the pterygoid plate.

Implant superstructure: In order to construct the prosthetic structure, the plastic UCLAs (Sistema Nacional de Implantes, São Paulo, Brazil) were attached directly to the implants. A straight DuraLay (Reliance Dental Mfg Inc., Illinois, USA) bar was obtained and connected to the UCLAs using a flat surface and a bubble level tool. Three equidistant wholes were created in the structure to adapt the adjustable ends of the universal test machine (EMIC – DL 3000 model) and an optimal force distribution. This piece was sent to a prosthetic laboratory for integration and conversion into a cobalt-chromium alloy. A prosthetic torque meter (Sistema Nacional de Implantes, São Paulo, Brazil) calibrated to 20 N was used to connect the metallic structure to the implants with titanium screws and the resulting adaptation was observed.

### Extensometric Analysis

**Figure 1:**
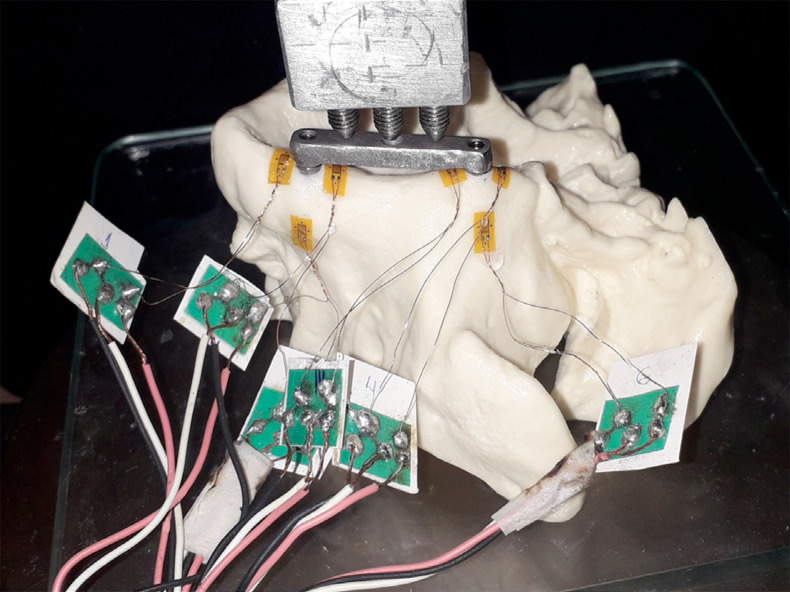
Strain gauges connected to the bridge and model attached to the test machine – control group.

Five replicas of each rehabilitation protocol were manufactured using polyurethane (Nacional Ossos, Jaú, Brazil) with six linear gauges and grid dimensions of 2.0 X 1.5 mm (PA-06-060BA-350 - Excel Sensores Com. e Exp., São Paulo, Brazil) attached horizontally with cyanoacrylate (Super Bonder, Loctite, Henkel Ltda, São Paulo, Brazil) at the apical anterior region (Aa), mesial anterior region (Ma), distal anterior region (Da), apical posterior region (Ap), mesial posterior region (Mp), and distal posterior region (Dp) at a distance of 2 mm of each implant (Figure 1). The wires from the strain gauges were connected to a multichannel bridge amplifier, thereby forming one arm of the bridge (Figure 1). The models were positioned in the universal test machine, and data were collected prior to loading with no records observed. Five cycles of repeated axial tensile loading (100 N) were performed in the three equidistant points over the superstructure at 0.5 mm/min. A computer was interfaced with the amplifier in order to record the output signal of the polyurethane surface. The data were then acquired using the acquisition system software (System 5000 Model 5100B; Vishay, Malvern, USA). The mean stress data from all sites was entered into an Excel spreadsheet for analysis (Microsoft Office Excel, Redmond, USA).

### Photoelastic analysis

The two configurations were manufactured (Nacional Ossos, Jaú, Brazil) using photoelastic resin (Araldite, Huntsman, The Woodlands, USA) in accordance with the instructions provided by the manufacturer for the polymerization process. During this process, the fixtures were incorporated into the photoelastic analysis. The models were positioned in the universal test machine (EMIC-DL 3000 model) and subjected to axial tensile loading (100 N)^29,31^ at three equidistant points over the superstructure at a rate of 0.5 mm/min. The stress due to the presence of the implant in the model was confirmed using a circular polariscope, and photographs were taken before and after loading. These were then visualized in a computer graphics program (Adobe Photoshop CS6, Adobe Systems, San Jose, CA, USA) to facilitate analysis.

**Figure 2:**
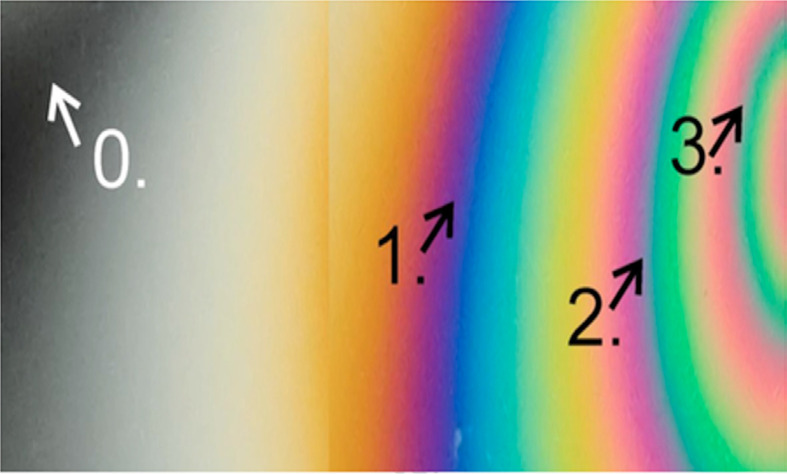
Fringe order and the corresponding stress value: 0 (black), 1 (violet/blue transition), 2 (purple/blue transition), and 3 (red/green transition).

Stress distribution was observed through the isochromatic fringes, counting each fringe order as it was passed (Figure 2): 0 (black), 1 (violet/blue transition), 2 (purple/blue transition), and 3 (red/green transition). The analysis was also divided according to the number of fringes of high intensity and the area of stress concentration^29^. All images were evaluated by the same operator.

The data were submitted to the SigmaPlot software (SigmaPlot software, version 12.0, 2011, San Jose, USA) and analyzed according to data distribution employing both the Shapiro-Wilk test and equality of variance. Subsequently, the data set was classified according to the variables. A Student’s t-test was used when the data exhibited a normal distribution of variances, whereas a Mann-Whitney U test was used for data sets with a non-normal distribution. A significance level of 0.5% was established.

## Results

### Extensometric Analysis

The mean values and standard deviations of the range of stress dissipation of each region calculated over five loading cycles indicated good repeatability of the measurements and are listed in Table 1. The comparison between the control and pterygoid models revealed no statistically significant differences (p = 0.37). Similarly, the comparison between the anterior and posterior implants in isolation yielded no statistically significant results (p = 0.08 and 0.74, respectively).

**Table 1:** Deformation values in the anterior implant by region.

Numeric Evaluation of Mean Strain (Standard Deviation) - Anterior Implant			
Group	Mesial	Distal	Apical
Control	49.596 (12.554)	341.82 (317.173)	544.069 (202.025)
Pterygoid	57.591 (16.128)	260 (236.56)	265.826 (60.894)
p-value	0.407	0.008	0.018

The extensometer analysis revealed that the highest mean force dissipation was observed in the posterior implant in the pterygoid proposal and in two of three regions of the posterior implant in the control models. In the pterygoid group, the highest numbers were observed at Ap, followed by Dp, Mp, and Aa. The lowest values were found in Ma and Da. The control group also presented the highest numbers in Ap, followed by Aa, Mp, and Dp. Similar to the pterygoid configuration, the lowest mean values were seen in Ma and Da (Table 1).

The pterygoid models exhibited a statistically significant reduction in mean stress dissipation in the Ap, Da, Aa, and Dp (p ≤ 0.001, 0.008, 0.018, and 0.027, respectively) compared to the control group. No significant differences were observed in the values at the other sites between the two rehabilitation models (Table 1).

### Photoelastic Analysis

**Figure 3:**
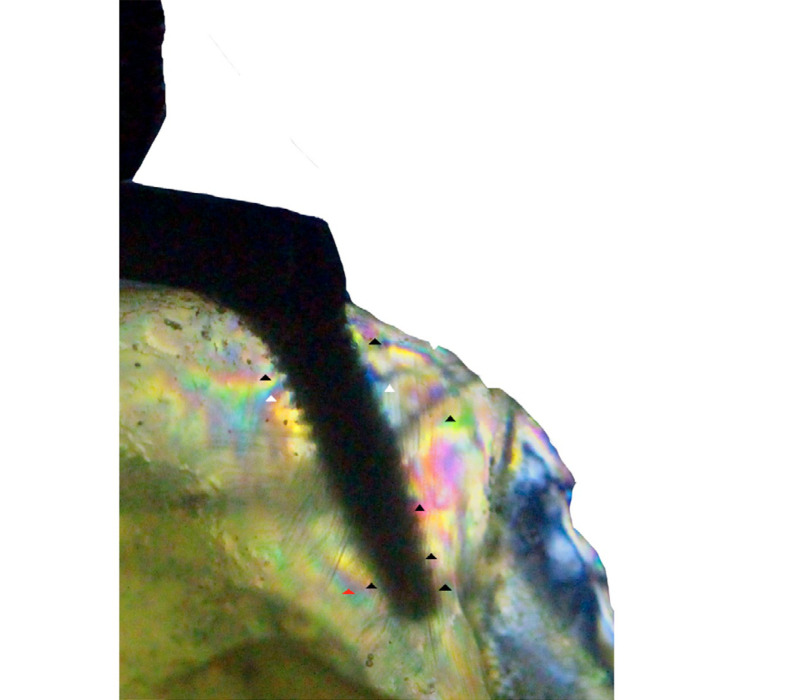
Isochromatic fringes around a distal implant – Pterygoid model (low intensity – white arrow; medium intensity – red arrow; high intensity – black arrow).

In the pterygoid group, order 1 fringes, which indicate low stress, were observed in the cervical mesial and distal regions (white arrows). Order 2 fringes were identified in the apical region of the mesial surface (red arrow) and order 3 fringes (black arrows) were visualized at the cervical-distal and apical regions at a higher concentration also present at the apex (Figure 3, Table 2).

**Table 2:** Deformation values in the posterior implant by region.

Numeric evaluation of mean strain (standard deviation) - Anterior implant			
Group	Mesial	Distal	Apical
Control	485.479 (81.532)	370.006 (67.239)	1616.786 (132.424)
Pterygoid	447.171 (69.379)	591.425 (171.002)	693.179 (53.292)
p-value	0.447	0.027	< 0.001

**Figure 4:**
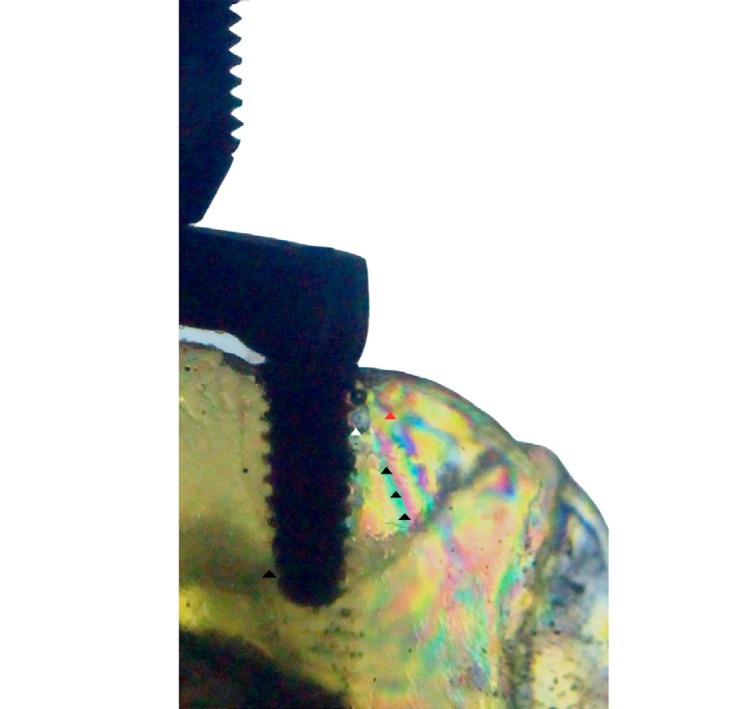
Isochromatic fringes around a distal implant – Control model (low intensity – white arrow; medium intensity – red arrow; high intensity – black arrow).

**Figure 5:**
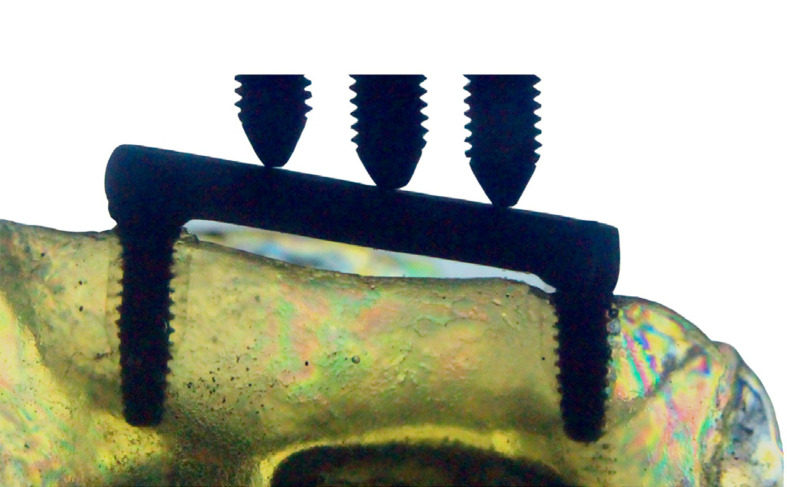
Photoelastic test – Control model.

**Figure 6:**
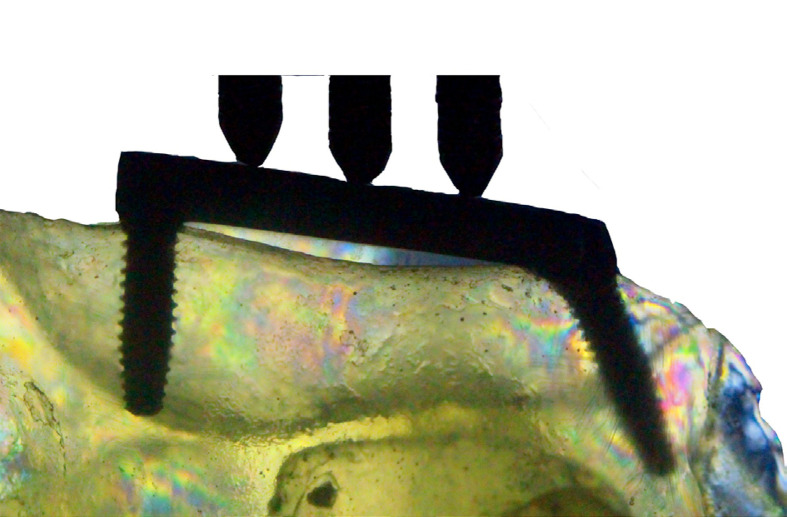
Photoelastic test – Pterygoid model.

In the control group, order 1 fringes (white arrow) were observed at low concentrations at the distal-cervical region of the posterior implant. Additionally, fringes of low concentration (red arrow) were observed at the distal end of the cervical surface, as well as high-intensity fringes (black arrows) at the apical surface (Figure 4, Table 2). No fringes were observed in the vicinity of the anterior implants in either configuration (Figures 5 and 6).

## Discussion

Pterygoid implants are a potential alternative for rehabilitating an atrophic maxilla.^3,5-6,24-27^ In this modality, an implant is inserted into the pillar, which consists of the maxillary tuberosity, the pyramidal process of the palatine bone, and the pterygoid process of the sphenoid bone. This provides support and stability for a bone-anchored prosthesis.^3,6,10,25,27^The technique has shown high success rates and survival, with a reduction in morbidity and a decrease in treatment time and cost when compared to other reconstruction procedures.^6,27^ From a biomechanical perspective, pterygoid implants do not require distal cantilevers, which avoids the inherent risks.^2,6,24,25,27^

Although well established, there is a paucity of mechanical tests to assess the stress behavior of a model of oral rehabilitation utilizing this technique.^33,34^ The objective of this study was to describe the load dissipation in models using a pterygoid implant and compare it to a control group employing axial implants. In clinical practice, both approaches are used to address the same situation and yield comparable prosthetic outcomes in posterior rehabilitation. However, there are notable differences in the inclination of the distal implant between the two models, with variations in their biomechanical behavior under loading. It is also evident that axial implants are not a viable option for use in an atrophic maxilla.

The survival rates of osseointegrated implants depend on the ability of the bone/implant interface or the prosthetic compound to dissipate stress adequately. Excessive loads and inadequate stress dissipation at the interface are critical issues that can affect the long-term success of the implant.^15,28-32^It is therefore crucial to understand the mechanics and load distribution involved in order to prevent implant failures and increase success rates.^30^ Over the past few decades, researchers have studied the biomechanical characteristics of implant-supported rehabilitations with the aim of elucidating the limits of force transmission and developing methods to evaluate the behavior of forces acting on the implant, prosthesis, prosthetic compounds, and bone.^15,28-32^

Authors^29^ proposed that a clinical evaluation would be the optimal method for analyzing the biomechanical response of dental implants. However, they considered this methodology to be practically infeasible due to the complex structures involved, which render direct clinical evaluation of intraosseous structures nearly impossible, and the ethical complexity of this type of study.^29,30^ In order to overcome these limitations, some in vitro methods employ finite element analysis, photoelasticity, and strain gauges for the evaluation the biomechanics of oral rehabilitations using osseointegrated implants.^15,28-34^These methods are complementary and cannot be considered superior to each another; rather, they are used in conjunction.^28,30^ In the present study, photoelasticity and extensometry were employed to demonstrate the biomechanical behavior of an experimental pterygoid model of oral rehabilitation, thereby facilitating qualitative and quantitative analysis.

Chen et al.^33^ employed finite element analysis to determine the best approach for pterygoid implant placement. The authors manufactured different models for rehabilitation purposes, utilizing four anterior and two pterygoid implants with 45 or 70-degree angle with the Frankfurt plane. The results demonstrated that the most angulated implants exhibited superior biomechanical performance. It was stated that load dissipation is more effectively transferred along the axis of the implant required to reach the pterygoid cortex, resulting in a more uniform distribution of forces. Furthermore, it was commented that pterygoid anchorage provides a better primary stability and is essential for osteointegration and immediate loading.^34^

In a study published in 2024, Vinodh et al. compared the efficacy of two models of rehabilitation with complete fixed dental prostheses. The authors highlighted the significant advancement in prosthetic rehabilitation of atrophic maxilla with the advent of the All-on-4 technique. However, by employing a strain gauge and finite element analysis, the researchers found a better load distribution and diminished stress in the models with pterygoid implants compared to the group with All-on-4 implants and prostheses with cantilever.^34^

It is necessary to consider certain aspects of the experimental model and interpret the results of this study with caution. The methodologies employed are subject to certain limitations, as they are used for estimating stress in models that cannot fully replicate the characteristics of human tissues.^28-30^ A photoelastic analysis provides only qualitative information.^30^ While a strain gauge can provide a complementary quantitative measurement, it can only be applied with accuracy on the external surface of a specimen and at a few locations due to space limitations.^33,36^

No significant difference in force distribution was found between models (p = 0.37). Similarly, an analysis of anterior and posterior implants in isolation between groups showed no statistically significant difference in force distribution (p = 0.08 and 0.74, respectively). Nevertheless, a greater dissipation of forces was observed in the vicinity of distal implants in both configurations, as evidenced by both quantitative and qualitative methods. The apical region of both models exhibited the highest values of stress dissipation in the extensometric analysis, with 1616.7 and 696.1 in the control and pterygoid groups, respectively. Additionally, the photoelastic test revealed elevated stress concentrations at the apex, particularly near the pterygoid implant. These findings are of interest from a clinical perspective, given that the apical portion of pterygoid implants is anchored in the cortical bone.^6,10,24-25,34^

As observed in other mechanical studies, an additional limitation of our study was the direction of the applied forces.^33-36^ While vertical loads were applied to three equidistant points corresponding to the second premolar, first and second molars, in agreement with previous studies that show higher forces in the occlusal region of the molar teeth, the static vertical loads used in this study do not reproduce the human masticatory movements. Furthermore, the absence of oblique forces could narrow the generalizability of our results.^6,36^

The extensometric analysis revealed low microstrain values in two out of the three strain gauges positioned around the pterygoid implant. Significant differences were observed in the distal and apical regions when compared to the control group (p = 0.018 and < 0.001, respectively). Nevertheless, the lowest values were observed in the control group at the distal surface of the posterior implant (p = 0.027). In the photoelasticity analysis, high-intensity fringes were observed in the cervical-distal region of the pterygoid implant. These findings align with those of Cidade et al.^38^ and Goiato et al.,^39^ who demonstrated elevated stress levels at the distal surface of angled or pre-angled single implants, with an increasing number of fringes corresponding to the implant’s highest inclination under axial forces. Elsyad et al.^40^ also observed comparable outcomes in a study utilizing strain gauges, with higher stress levels correlating with increased implant inclination.

The mechanical tests conducted in the present study also demonstrated that the highest forces were dissipated in the apical portion of the posterior implant in both experimental groups. The extensometric analysis revealed considerably higher values in the control group, which can be attributed to the axial forces travelling along the implant axis and dissipating in the apical area of parallel implants.^28,41^ Furthermore, it can be posited that the optimal performance observed in the apical zone of the pterygoid group may be attributed to the predominant application of forces to the pterygoid plate, as evidenced by the photoelasticity data, with a comparatively lower distribution across other regions. However, due to the narrow anatomy of the pterygoid plate, it was not possible to install a strain gauge in this structure in order to obtain measurements.

A quantitative and qualitative mechanical analysis revealed a high distribution of forces in the apical areas of both models. Both groups exhibited elevated stress values and the formation of fringes. The utilization of pterygoid implants may prove clinically advantageous, given the presence of cortical bone for anchorage. It is recommended that future research be conducted using scale models with strain gauges attached to the pterygoid plate, given the inability to measure the peak value in the structure.

## Conclusion

Despite several limitations due to the experimental model, this paper aimed to demonstrate the biomechanical behavior of a pterygoid implant for oral rehabilitation. The strain gauge analysis revealed that the highest values were observed in the apical regions of the posterior implants (control: 1616.79 vs. pterygoid: 693.18; p<0.001), while the lowest values were noted in the mesial region of the anterior implant (control: 49.60 vs. pterygoid: 47.59; p=0.40). The use of photoelasticity revealed the presence of high-intensity isochromatic fringes at the apex of the axial implant in the control model, as well as in the cervical-distal and apical regions of the pterygoid implant, where a high concentration was also observed. Although no statistically significant results were obtained from the quantitative analysis, our findings suggest that the favorable outcomes observed in clinical studies are due to the high force dissipation to the pterygoid plate, which consists of dense cortical bone.
